# Overcoming the loss of blue sensitivity through opsin duplication in the largest animal group, beetles

**DOI:** 10.1038/s41598-017-00061-7

**Published:** 2017-01-31

**Authors:** Camilla R. Sharkey, M. Stanley Fujimoto, Nathan P. Lord, Seunggwan Shin, Duane D. McKenna, Anton Suvorov, Gavin J. Martin, Seth M. Bybee

**Affiliations:** 1 Department of Biology, Brigham Young University, 4102 LSB, Provo, UT 84602 USA; 2 Computer Science Department, Brigham Young University, Provo, Utah 84602 USA; 3 Department of Biological and Environmental Sciences, Georgia College & State University, Campus Box 081, Milledgeville, GA 31061 USA; 4 Department of Biological Sciences, University of Memphis, 3700 Walker Avenue, Memphis, TN 38152 USA

## Abstract

Opsin proteins are fundamental components of animal vision whose structure largely determines the sensitivity of visual pigments to different wavelengths of light. Surprisingly little is known about opsin evolution in beetles, even though they are the most species rich animal group on Earth and exhibit considerable variation in visual system sensitivities. We reveal the patterns of opsin evolution across 62 beetle species and relatives. Our results show that the major insect opsin class (SW) that typically confers sensitivity to “blue” wavelengths was lost ~300 million years ago, before the origin of modern beetles. We propose that UV and LW opsin gene duplications have restored the potential for trichromacy (three separate channels for colour vision) in beetles up to 12 times and more specifically, duplications within the UV opsin class have likely led to the restoration of “blue” sensitivity up to 10 times. This finding reveals unexpected plasticity within the insect visual system and highlights its remarkable ability to evolve and adapt to the available light and visual cues present in the environment.

## Introduction

At the molecular level, the wavelength sensitivity of an animal photoreceptor is determined by the photopigment, comprising an opsin protein bound to a light-absorbing chromophore. Insects commonly possess three opsin proteins (UV, SW and LW) that form photopigments maximally sensitive to ultraviolet (~350 nm), blue (~440 nm) and green (~530 nm) wavelengths, respectively. As insect opsin genes form distinct phylogenetic clades according to their spectral class (UV, SW or LW) the sensitivity ranges of an insect visual system can usually be estimated by the complement of opsin genes present. In some insects photopigment sensitivity has extended outside of this range into the violet (~420 nm) and red (>600 nm) region of the light spectrum through duplications of the SW^1,2^ and LW opsins^2,3^, respectively.

Gene duplications occur at a proposed rate of 1 per 100 MY^4^ and for the majority of duplications, gene copies are lost within ~2 MY, through the accumulation of deleterious mutations^4^. However, gene copies may be retained if they acquire a novel function through sequence mutation (subfunctionalization)^4^ that leads to increased fitness. In the case of opsins, beneficial mutations in duplicates may lead to changes in photopigment properties, such as spectral sensitivity. Such duplications alongside losses of opsins have shaped the major animal opsin classes we observe today. Duplications of long-wavelength sensitive opsins are widespread across Arthropoda and are numerous in some lineages (e.g., up to 21 LW opsins in aeshnid dragonflies^2^, 25 in the genome of *Daphnia pulex* ^5^ and seven in mosquitoes^6^). UV duplications by contrast are rare and lineage specific, with only single duplication events reported in *Heliconius* butterflies^7^, planthoppers (Delphacidae)^8^, and two lineages of beetles^9,10^.

Molecular evidence suggests that the SW opsin class has been lost from a number of beetle lineages: fireflies (Lampyridae^11,12^), diving beetles (*Thermonectus marmoratus*)^9,13^, jewel beetles (Buprestidae)^10^ and darkling beetles (e.g., *Tribolium castaneum*)^14^. It has therefore been proposed that all beetles may lack the SW opsin class, which typically underpins visual sensitivity to blue wavelengths in insects. However, physiological evidence has revealed that a number of beetles do have blue sensitive photoreceptors: a ladybird (*Coccinella septempunctata*)^15,16^, a leaf beetle (*Leptinotarsa decemlineata* ^17^), two ground beetles (*Carabus* spp.)^18^ and a jewel beetle (*Agrilus planipennis*)^10^.

Two evolutionary scenarios are therefore possible. Firstly, the SW opsin class was lost independently in numerous beetle lineages, or secondly, this opsin class was lost prior to or during the early evolutionary history of beetles and blue light sensitivity was regained multiple times, independently of the ancestral SW opsin. Due to the paucity of studies on opsins from beetles and related groups, the timings of the SW opsin losses remain unclear. To address this, we examined the opsin repertoire of a broad diversity of beetles representing most major lineages and included beetles with a wide range of life histories (e.g., diurnal, nocturnal, predaceous and pollinating beetles). Additionally, exemplars from the closely related orders Strepsiptera (twisted-wing parasites), Raphidioptera (snake flies), Megaloptera (alderflies, fishflies and dobsonflies) and Neuroptera (lacewings, antlions and mantidflies), comprising the other major lineages of the clade Neuropteroidea^19^ were also investigated.

## Results

### Loss of the SW opsin class

For this study, over two billion RNA-seq reads were assembled into more than six million gene transcripts (Table S1). In total, 204 opsins were recovered, of which 73% encode full-length proteins (Tables S2 and S3). In a homology search of 74 coleopteran transcriptomes across 29 families and 19 of 21 superfamilies, the SW opsin was not recovered. Furthermore, this opsin class was not detected in the 12 transcriptome assemblies from the other lineages of Neuropteroidea. While the majority of samples were derived from adult tissue (Table S1), larval tissue was also sequenced for 11 species. The SW opsin class was also not recovered from these samples, indicating that it has been lost in both major life stages. Similarly, both sexes were sequenced for 15 species, indicating that the SW opsin loss is not sex specific. Furthermore, we did not recover the SW opsin class from the genomes of four beetle species (Table S1). In all cases, there was no evidence for a SW opsin pseudogene but the absence of the SW gene in our analyses indicates that it has indeed been lost. We are confident that the phylogenetic breadth and quality of samples (Figure S1) used in this study was sufficient to rule out the possibility that this opsin class was present within Coleoptera but not recovered during our analysis. Bees, wasps and ants (Hymenoptera) are thought to be the closest relative of all remaining holometabolan insects, including the true files (Diptera), and moths and butterflies (Lepidoptera)^20^. The SW opsin has been retained in these orders^21–23^, implying that the loss of the SW opsin occurred in the lineage leading to Neuropteroidea, after this clade diverged from the rest of Holometabola, approximately 300 million years ago^19,24^.

### Opsin duplications

As the insect SW opsin class was lost prior to the radiation of Coleoptera, blue light sensitivity must have secondarily arisen in Coleoptera through an alternative mechanism to that of other insects. Recent evidence suggests that blue light sensitivity in jewel beetles (Buprestidae) has been achieved through duplication and subfunctionalization of the UV and/or LW opsin genes^10^. We performed homology searches for the presence of opsin gene duplications on 89 assembled neuropteroid transcriptomes, totalling 72 species. Phylogenetic analyses were performed to reconstruct species and opsin gene trees, which we then used to interpret the evolutionary history of opsin gene duplication events.

**Figure 1:**
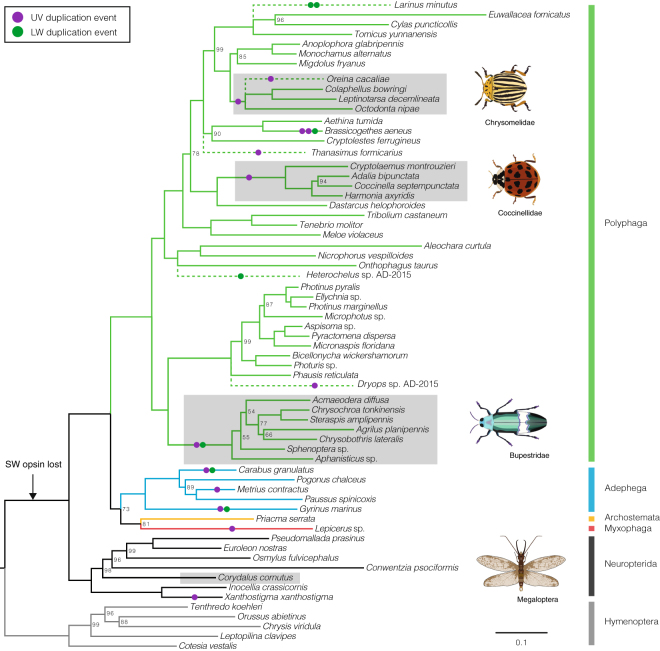
Opsin duplications within Coleoptera. Species phylogeny using 358 gene clusters from translated transcriptome assemblies, constructed using maximum likelihood. Node values indicate UFboot supports and only values <100 are shown. Strepsipterans are not included due to poor support and placement of these species. For the full topology see Figure S2. Three major coleopteran UV opsin clades are highlighted in grey. Additional species with opsin duplications that were not included in the original species phylogeny are included at proposed positions (dashed lines). Solid circles denote putative UV (purple) and LW (green) duplications. The proposed timing of the SW opsin class loss is indicated (black arrow).

**Figure 2:**
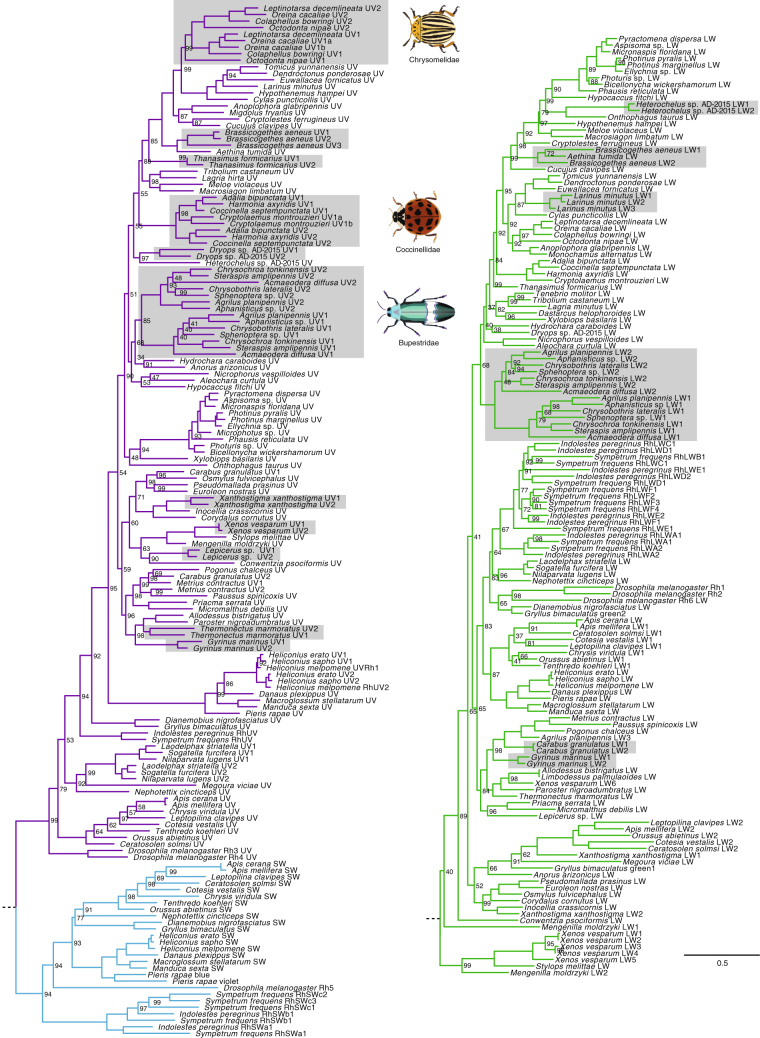
Beetle opsin gene phylogeny. Maximum likelihood DNA phylogeny of all visual opsin genes from this study and other insect UV (purple), SW (blue) and LW (green) opsins rooted to cephalopod opsins (not shown). Node values (UFBoot support values) are based on 10,000 replicates. Values of 100 are not shown. Duplicated neuropteroid opsins are highlighted in grey.

**Figure 3:**
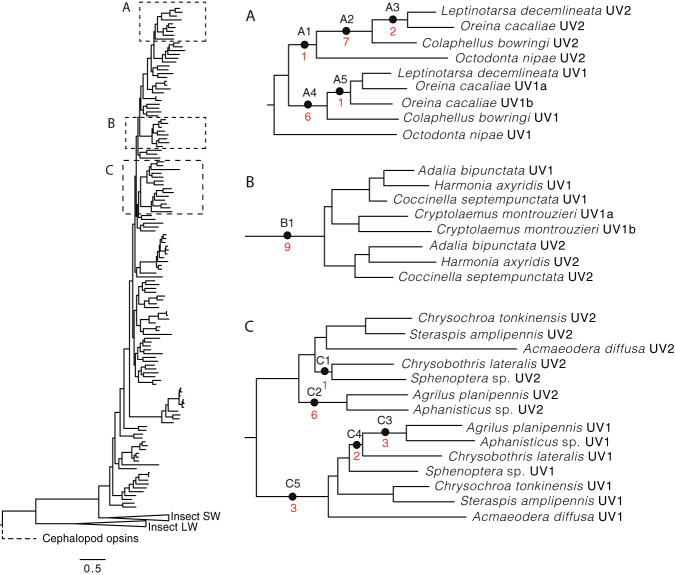
Signals of selection within beetle UV duplication clades. Branches (closed circles) and numbers of sites (red values) under positive selection in the three major coleopteran UV opsin clades. Positions of these clades, (**A**) (Chrysomelidae), (**B**) (Coccinellidae) and (**C**) (Buprestidae), on the full opsin DNA phylogeny are indicated (dashed boxes). For further positive selection statistics and amino acid sites see Tables S4 and S5.

**Figure 4:**
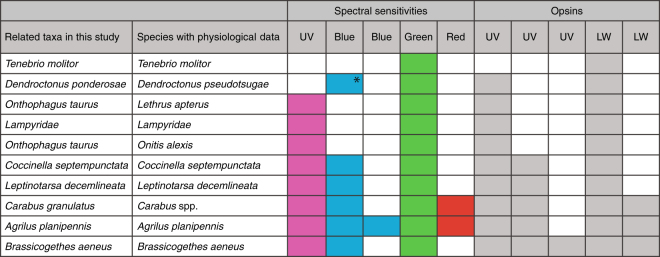
Comparison of spectral sensitivities and opsins found in this study for matching or similar species. *Measurements from *Dendroctonus pseudotsugae* were made in the absence of UV stimuli. The “blue-sensitive” photoreceptors discovered in this species most likely peak in the UV.

We recovered UV duplications from three of the four coleopteran suborders: from the single species sampled from the suborder Myxophaga (*Lepicerus* sp.), three of five species from the suborder Adephega and 18 of 54 species from the suborder Polyphaga (Fig. 1; Table S2). UV duplications were not recovered in either species from suborder Archostemata (*Priacma serrata* and *Micromalthus debilis*) (Table S2). In three cases, the phylogenetic placement of both UV copies into separate monophyletic clades in the gene tree reconstructed from DNA sequences (Figs 2 and 3) suggests that opsin gene duplications occurred prior to the crown diversification of leaf beetles (Chrysomelidae), ladybirds (Coccinellidae) and jewel beetles (Buprestidae) (Fig. 2). Ancestral states of opsin duplication were reconstructed on the species phylogeny, to infer likely patterns of duplication across Coleoptera. Parsimony and maximum likelihood reconstructions (Figure S3), alongside the phylogenetic placement of opsin duplicates along independent lineages, suggest that many separate opsin gene duplication events occurred within Coleoptera. In total, including the previously described *Thermonectus marmoratus* and Buprestidae duplications, our results are consistent with the existence of 12 independent UV opsin duplications, two of which occurred in one species, the pollen beetle *Brassicogethes aeneus* (Nitidulidae) (Fig. 1). UV opsins are absent in three beetle species, *Dastarcus helophoroides*, *Monochamus alternatus* and *Tenebrio molitor*, suggesting low UV opsin gene expression or a lack of UV opsins. Physiological evidence also supports a lack of UV sensitivity in *T. molitor* (Fig. 4)^25^. In the remaining neuropteroid orders, UV opsin duplications were observed in snakeflies (Raphidioptera) and one in twisted-wing parasites (Strepsiptera) (Figs 1 and 2).

Novel LW opsin gene duplications were discovered in five coleopteran species across five families: the flower weevil *Larinus minutus* (Curculionidae), the whirligig beetle *Gyrinus marinus* (Gyrinidae), the pollen beetle *Brassicogethes aeneus* (Nitidulidae), the monkey beetle *Heterochelus* sp. (Scarabaeidae) and the ground beetle *Carabus granulatus* (Carabidae). The DNA sequences of *Carabus granulatus* LW opsins were identical at the protein level, however, as the full length LW2 was not recovered in this species (137 amino acids; Table S3), it remains to be examined how distinct the full-length copies of these two opsins are. One additional buprestid (*Aphanisticus* sp.) was also found to have two LW opsin copies, confirming previous findings from six other buprestid species^10^. LW duplications are also present in two of three Strepsiptera species with *Xenos vesparum* possessing five unique LW opsin proteins.

### Sites under selection

To explore potential amino acid sites responsible for spectral shifts in beetle UV opsins with putative blue light sensitivity, we tested branches in the opsin gene tree (Fig. 2) and individual amino acid sites for positive selection (PS). This was carried out in the three major clades of beetle UV opsins duplicates that have complementary physiological evidence of blue-sensitive photoreceptors (Buprestidae, Coccinellidae and Chrysomelidae). Site selection analyses reveal only four sites under PS adjacent to the chromophore-binding pocket, and the recovered positively selected sites were not congruent between lineages (Fig. 3; Table S4). It is therefore difficult to hypothesise about the potential specific shifting mechanisms behind photopigment spectral diversity in these groups. Interestingly, however, all coccinellid, two chrysomelid and one buprestid UV opsin duplicates have hallmarks of insect SW opsins (Tables S4 and S5). This includes a neutral or negatively charged amino acid rather than positively charged lysine (K) at bovine site 90 and phenylalanine (F) replacing tyrosine (Y) at bovine site 113^26^. Replacing K with a neutral amino acid at site 90 in the *Drosophila* Rh3 UV opsin, shifts sensitivity by 73 nm into the blue wavelengths^26^. This single amino acid substitution may be sufficient to render a number of beetle UV photopigments found in this study (*Harmonia axyridis* UV2, *Leptinotarsa decemlineata* UV2, *Octodonta nipae*, UV2 *Oreina cacaliae* UV2, *Steraspis amplipennis* UV1) functionally blue sensitive. Little is known about the spectral shifting mechanisms across Coleoptera^10,12,27^ but our findings, coupled with physiological evidence for photoreceptor diversity, highlight beetles as a highly attractive group for future studies.

## Discussion

Opsin losses have occurred in other animals, including the American cockroach (*Periplaneta americana*)^28^, deep sea fish^29^, fossorial snakes^30^, caecilians^31^ and both nocturnal and aquatic mammals^32–34^. Such losses are typically associated with low-light or spectrally-attenuated environments. It has been proposed that the presumptive loss of tri- or di-chromatic colour vision under these conditions has little impact on fitness^35^ and due to the high cost of maintaining retinal tissue^36^, selection favours a reduction in visual system complexity (e.g., during the evolution of mammals)^35^. Furthermore, under these scotopic conditions, the saliency of wavelength specific cues is diminished^37^ and a system using two rather than three photoreceptor classes may be advantageous by increasing overall sensitivity to light^38^. Similar to what has been observed in other animal visual systems with reduced opsin diversity, it seems probable that the ancestor of Neuropteroidea was nocturnal or functioned at low light levels. Support for this hypothesis is also given by the preponderance of nocturnality (Neuroptera, Megaloptera, Raphidioptera) and evidence for visual function associated with nocturnality (Strepsiptera) in the other orders of Neuropteroidea^39,40^.

Opsin gene duplication and divergence is the primary mechanism by which novel photopigment sensitivities arise^41^. Throughout animal evolution, many instances of opsin gene duplications and losses have occurred, shaping the diverse repertoire of animal visual system sensitivities we observe today^41,42^. A well-known example is the duplication of the LWS opsin gene in Old World monkeys that has lead to increased discrimination between long wavelengths and therefore an increased ability to detect ripe fruits against a foliage background^43,44^. The distribution of opsin duplications across Coleoptera recovered in our analyses clearly show that opsin duplicates are not derived from an opsin-diverse common ancestor but, instead, appeared secondarily and independently in numerous lineages of beetles. The prevalence of highly divergent UV opsin duplicates across Coleoptera (Table S3) and the rarity of such duplicates in other animal visual systems suggests that there may be a greater selective advantage for additional UV opsins in beetles than other animals. It is highly unlikely that duplications reflect adult and larval opsin copies, as no difference in opsin copy number was found between the available adult and larval samples and the majority of samples used were derived from adult tissue (Table S1).

Spectral sensitivity data was available for six species included in this study, from two closely related *Carabus* species and one *Dendroctonus* species (Fig. 4)^16–18,25,27,45–49^. Comparing the number of opsin copies with photoreceptor sensitivities reveals that all instances of blue sensitivity co-occur with UV duplication (*Coccinella septempunctata*, *Leptinotarsa decemlineata*, *Brassicogethes aeneus*, *Agrilus planipennis* and *Carabus* spp.; Fig. 4). We propose that coleopteran UV duplications and subsequent amino acid changes serve to extend the sensitivity of the beetle visual system into the short or blue wavelengths, overcoming the ancestral loss of sensitivity to this region of the light spectrum and its presumed limitations on spectral sensitivity and discrimination in diurnal and other comparatively high-light environments.

We found that beetle species with additional photoreceptor sensitivities had extra opsin copies (Fig. 4) suggesting duplication and subfunctionalization as a route for the evolution of novel photopigment sensitivities. If the evolution of beetle opsin duplicates has been shaped by visual ecology, one might expect to find a clear link between opsin diversity and the use of visual cues or visual environment. Indeed, duplications are widespread amongst known diurnal species and those with eye morphologies that are indicative of activity in high light environments (e.g., large compound eyes, high density of corneal pigments, apposition-type eye structure)^50^. Opsin duplications were predominantly found in species with behaviours often guided by visual cues, and in many of these species (see references), vision has been shown to be the primary cue for such behaviours: flower visitation (*Larinus minutus*, *Brassicogethes aeneus* ^51^ and *Heterochelus* sp.^52,53^), predation (coccinellids^54^, gyrinids^55,56^, *Thanasimus formicarius*, *Carabus granulatus*, *Metrius contractus*), host plant detection (chrysomelids^57,58^, coccinellids^59^), and mate recognition (buprestids^60^). Duplications were notably absent in nocturnal species, with the exception of the nocturnal active predator, *Carabus granulatus*, which has been shown to possess a number of spectrally distinct photoreceptors^18^.

As is the case in butterflies and moths (Lepidoptera)^61^ and dragon/damselflies (Odonata)^2,62^, beetle LW opsin duplications may serve to extend sensitivity into the longer wavelengths. However, further physiological measurements from beetle species with LW duplications are necessary to confirm this. Interestingly, whilst LW duplications are less common than UV duplications within Coleoptera, they are ubiquitous amongst flower-visiting lineages: *Brassicogethes aeneus* (Nitidulidae: 3 UV and 2 LW), *Heterochelus* sp. (Scarabaeidae: 1 UV and 2 LW) and the weevil, *Larinus minutus* (Curculionidae: 1 UV and 3 LW). This points towards a potential role for extended long wavelength sensitivity to locate flowers. A recent study found long wavelength-shifted photoreceptors (628 nm) in the flower-pollinating scarab, *Pygopleurus israelitus*, which is likely an adaptation to enhance the detection of red and orange flowers^63^. Physiological data from the red palm weevil, *Rynchophorus ferrugineus*, also reveals additional long wavelength-shifted photoreceptors^64^. The pollen beetle, *B. aeneus*, with 3 UV and 2 LW opsins, has the highest molecular complexity of opsins amongst all beetles studied thus far.

Our study reveals that some groups of beetles may have spectral sensitivity capabilities that match or even exceed those of pollinating bees and wasps (Hymenoptera), particularly in the long wavelengths. Traditionally it was assumed that trichromatic insect pollinators shaped the evolution of flower visual signals. Interestingly, our results suggest that beetle pollinators secondarily evolved the potential for trichromacy alongside the evolution of angiosperms. As beetles were presumably some of the first pollinating insects, this raises the interesting question of whether or not colour vision in beetles co-evolved with flowers or whether the beetle visual system adapted to detect flowers whose visual signals had already evolved alongside trichromatic pollinations, such as Hymenoptera.

## Methods

### Samples used

All available neuropteroid RNA sequence reads were obtained from the Sequence Read Archive (SRA) and raw RNA-seq data were included from refs 10 and 11 (Table S1). Data for an additional buprestid species (*Aphanisticus* sp.) were also generated (see ref. 10 for methods). Trimmomatic (v0.32)^65^ was used to trim raw sequence reads of Illumina-specific adapters followed by gentle trimming of low-quality bases (Phred score <5) from the start and ends of reads according to refs 66 and 67. Finally, all reads with a resulting minimum length of <25 bases were removed. The resulting reads were assembled using Trinity (v2.1.1)^68^ with default parameters. Thirteen additional unpublished assemblies were provided by 1KITE (see Tables S1 and S6). For these samples, RNA extraction and sequencing was carried out as described in ref. 24. Details regarding assembly and steps to remove cross-contamination are outlined in ref. 69. Transcriptome assemblies have been deposited into the NCBI TSA database (see Table S6 for accession, Bioproject IDs and assembly versions).

### Assembly quality quantification

To ensure that all whole-body transcriptomes were of high quality for opsin detection, the completeness of each assembly was estimated using orthology detection (BUSCO v1.1b1)^70^ using default settings to determine the presence of 2675 arthropod Benchmarking Universal Single-Copy Orthologs (BUSCOs; Benchmarking Universal Single-Copy Orthologs). The proportion of genes detected was used as an indicator of coverage for each transcriptome. Additionally, genes from the insect phototransduction pathway were extracted from each assembly, using a database of photransduction gene proteins, obtained from the genomes of 10 insect species, including one coleopteran (*Tribolium castaneum*, *Acyrthosiphon pisum*, *Pediculus humanus corporis*, *Nasonia vitripennis*, *Solenopsis invicta*, *Aedes aegypti*, *Culex quinquefasciatus*, *Plutella xylostella*, *Apis mellifera*, *Camponotus floridanus*; KEGG ID tca04745). Coding regions within the assemblies were predicted using TransDecoder (http://transdecoder.github.io), which retains the longest open reading frame (ORF). To ensure that no putative phototransduction genes were discarded prematurely, all predicted ORFs were also searched against a BLAST database of the phototransduction genes using blastp (BLAST+ v2.2.31)^71^ with e-value threshold of 0.001. The resulting ORF dataset was then searched against an HMM database of the phototransduction genes using hmmscan implemented in HMMER (v3.1b2)^72^. Assembly statistics, including total contigs recovered and N50 values, were used alongside the number of phototransduction genes present and the quality of opsins recovered (see below) to assess transcriptome quality. After examining all criteria, assemblies with fewer than 10 of 15 phototransduction gene orthologs were removed prior to further analysis.

### Opsin extraction

Opsins were extracted from all remaining assemblies (74 Coleoptera, four Neuroptera, five Strepsiptera, two Raphidioptera, one Megaloptera and five Hymenoptera; Table S1). Opsins were extracted using the same methods used for the detection of phototransduction genes (see above) but instead using a database of known arthropod opsins (orthoDB EOG8NKF98) plus full-length coleopteran opsin genes from Lampyridae and *Thermonectus marmoratus*. To ensure that all available opsin copies were extracted, assemblies were secondarily analysed using an additional pipeline (PIA)^73^. All sequences were also BLASTed (https://blast.ncbi.nlm.nih.gov/) and subject to phylogenetic analysis to confirm that they were visual opsin proteins. Many of the samples contained tissue from multiple individuals, therefore, to exclude inter-specific variation in opsin gene copy, highly similar opsin duplicates (>99% sequence identity) were removed (CD-hit v4.6.4)^74,75^. Sequence identity was approximated for the remaining opsin duplicates using CD-hit (v4.6.4) and BLAST. Opsin sequences have been deposited in GenBank with accession numbers KY368182–KY368379. Opsins were also obtained from four coleopterans using a tBLASTn search of coleopteran opsins against *Tribolium castaneum* (Tenebrionidae), *Anoplophora glabripennis* (Cerambycidae)^76^, *Dendroctonus ponderosae* and *Hypothenemus hampei* (Curculionidae) genomes (Table S1).

### Opsin phylogeny

Putative nucleotide opsin sequences were subject to a codon alignment using MAFFT (v7.273)^77^ with 98 insect opsin sequences, and five outgroup cephalopod opsins (see Table S7 for accession numbers) specifying automatic alignment strategy selection. All coleopteran Rh7 opsins, of which the function is unknown, were excluded to reduce the number of alignment gaps. Potential contaminate opsin sequences and non-visual opsins (peropsins and RGRs) were also removed. Phylogenetic inference was performed on all final opsin nucleotide sequences using maximum likelihood (IQ-TREE v1.4.1)^78^ with 10,000 UFBoot iterations. Estimation of the best fitting model was carried out using ModelFinder within IQ-TREE. LG + F + I + G4 was selected as the best-fit model. All trees were edited in FigTree (v.1.4.2 http://tree.bio.ed.ac.uk/software/figtree/).

### Species phylogeny

The 13 transcriptome assemblies provided by 1KITE were excluded from the species phylogeny according to 1KITE’s data release policy. To construct a species phylogeny, protein ORFs were generated from the remaining transcriptomes, using only one per species, with male adult samples used when possible. Orthologous gene clusters were generated using OrthoMCL (v2.0.9)^79^ and individually aligned using MAFFT (v7.273)^77^ with automatic alignment strategy selection. Each cluster represented a single gene and for species with multiple isoforms per gene, only the longest isoform was retained. Clusters were then filtered using a machine-learning algorithm implemented in OGCleaner^80,81^ to remove low quality putative homology clusters. Alignment quality was assessed using Aliscore (v2.0)^82^, and Alicut (v2.0) was used to remove ambiguously aligned positions in the multiple sequence alignments^82,83^. Only clusters with genes from at least 20% of species were used for further analysis. Lastly, a supermatrix was generated of 358 aligned gene clusters, which was used to infer a maximum-likelihood species phylogeny (IQ-TREE)^78^ with 10,000 ultrafast bootstrap (UFBoot) iterations^84^. UV and LW opsin duplication events were reconstructed on the species phylogeny, using both parsimony and maximum likelihood frameworks for ancestral state reconstruction in Mesquite (v3.04)^85^. Likelihood was estimated using the Markov k-state 1 (Mk1) parameter model, whereby all character changes are equally probable. Two characters were specified according to the opsin gene data: single opsin gene or duplicated opsin. Multiple duplications within one species were assigned the latter category. Both ancestral state reconstruction and the topology of the opsin gene tree were used to infer the pattern of duplications across Coleoptera.

### Positive selection analysis

Selection analysis was performed on the maximum likelihood topology using a full length DNA alignment of all opsins in this study, 98 additional insect opsins and five cephalopod opsins (Table S7). To test for signals of episodic positive selection (PS), we used CodeML within PAML (v4.9a)^86^. The branch-site model A, which allows ω variation among sites as well as tree branches, was used to detect signatures of PS affecting certain lineages and sites^87^. In order to determine significance of inferred selection patterns, we compared the branch-site null model A, which assumes fixed ω = 1 (i.e. neutrality) against the branch-site model A. Then, the test statistic for likelihood ratio test (LRT) was calculated as twice the difference in log likelihood (ℓ) between the two models (2Δℓ). Significance was assessed using a chi-square distribution with one degree of freedom. To avoid local optima, starting values of 0.1, 1 and 2 ω were used and the result with the best ℓ retained. A Bayes empirical Bayes approach was taken to determine amino acid sites likely to be under PS according a posterior probability with significance at ≥95%. The position of the chromophore-binding pocket (i.e. the region of the protein that interacts with the chromophore) was identified using 3D protein modelling (I-TASSER online server)^88,89^ using squid rhodopsin as a template (PDB model 2Z73A)^90^. Sites within the binding pocket were highlighted as potential candidates for spectral tuning and compared with known tuning sites in other taxa.

## Electronic supplementary material

Supplementary Information
