# Supplementary material for: Overcoming the loss of blue sensitivity through opsin duplication in the largest animal group, beetles
Source: Sci Rep. 2017 Jan 31;7:8. doi: 10.1038/s41598-017-00061-7 (PMC5428366; doi:10.1038/s41598-017-00061-7)
Supplement: Supplementary file 1 — Supplementary Information [file 41598_2017_61_MOESM1_ESM.pdf]

Overcoming the loss of blue sensitivity through opsin duplication in the largest animal group, beetles

Camilla R. Sharkey*[1], M. Stanley Fujimoto[2], Nathan P. Lord[3], Seunggwan Shin[4], Duane D. McKenna[4], Anton Suvorov[1], Gavin J. Martin[1], Seth M. Bybee[1]

[1] Department of Biology, Brigham Young University, 4102 LSB, Provo, UT 84602, USA.
[2] Computer Science Department, Brigham Young University, Provo, Utah 84602, USA.
[3] Department of Biological and Environmental Sciences, Georgia College & State University, Campus Box 081, Milledgeville, GA 31061, USA.
[4] Department of Biological Sciences, University of Memphis, 3700 Walker Avenue, Memphis, TN 38152, USA.

*Correspondence: camilla.r.sharkey@gmail.com

Supplementary Data

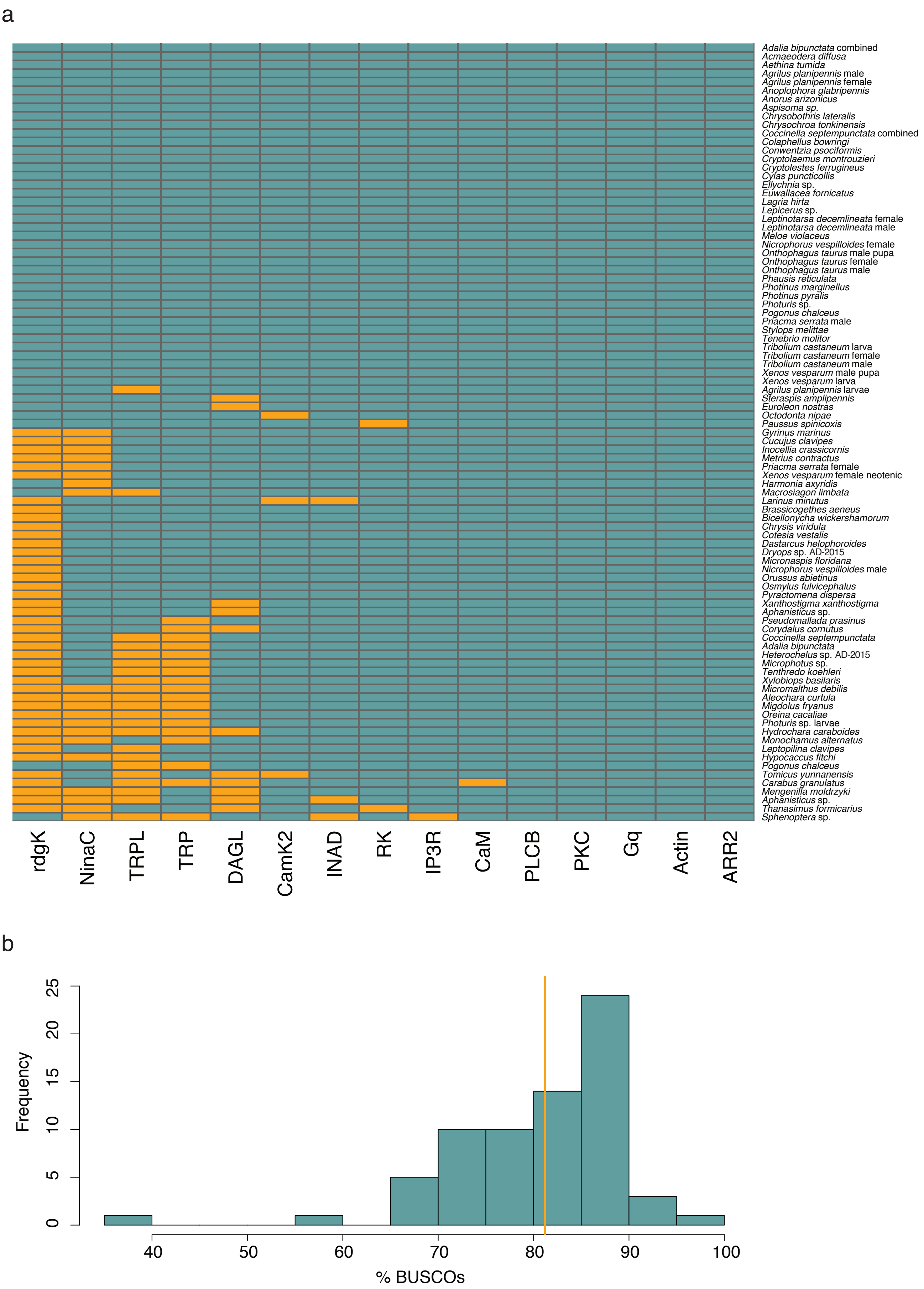

Figure S1. a: Heatmap showing the presence (blue) or absence (orange) of 15 insect phototransduction genes for each transcriptome assembly used in this study. Orthologs were detected at a conservative threshold of e-100.
b: The distribution of BUSCOs (Benchmarking Universal Single-Copy Orthologs) across all whole-body samples and the mean value (orange line). Higher values denote a larger proportion of BUSCOs present and therefore indicate a more complete transcriptome.

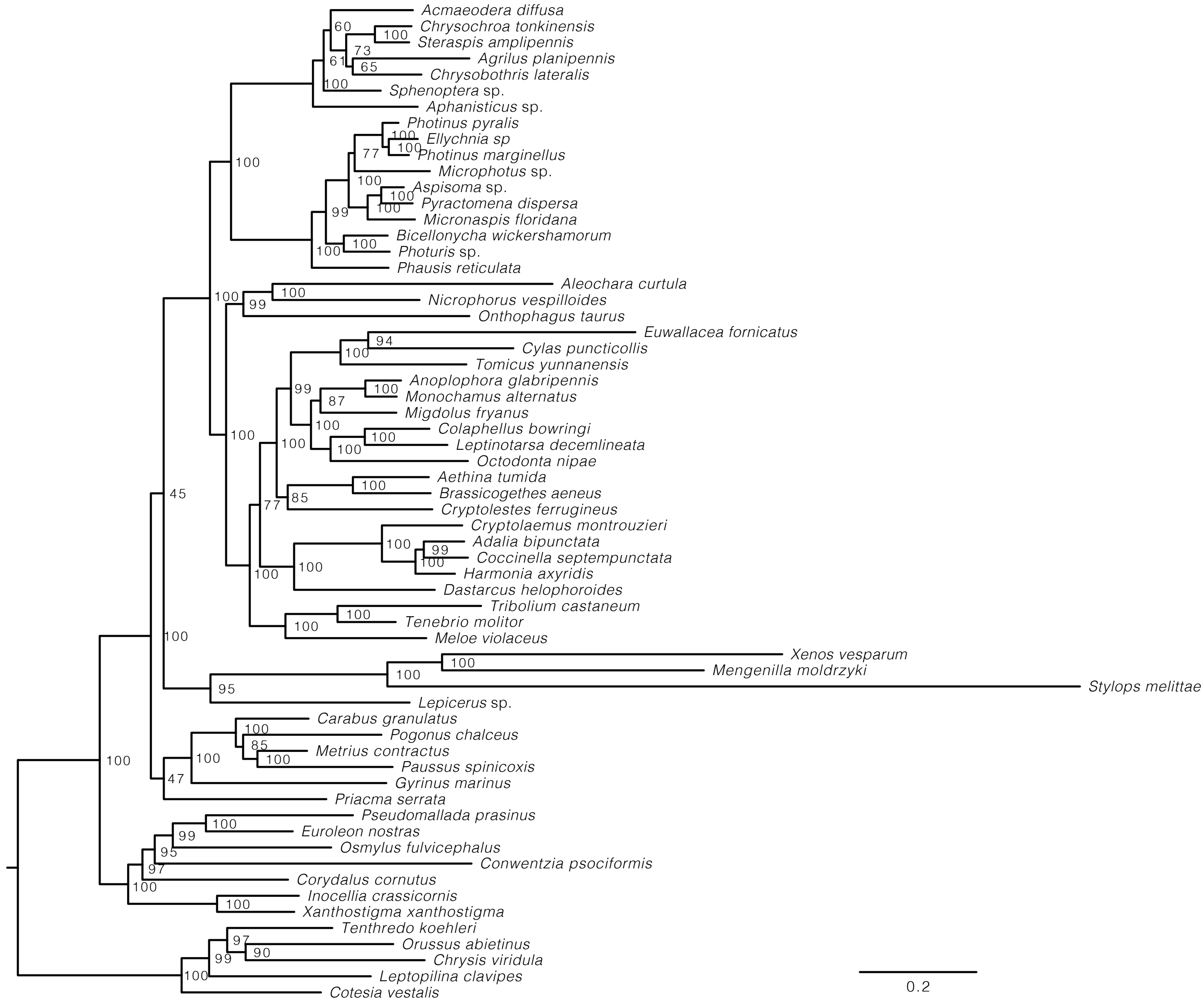

Figure S2. Full maximum-likelihood species phylogeny, including poorly supported strepsipterans (*Xenos* sp., *Mengenilla* sp. and *Stylops* sp.), using 358 gene clusters from translated transcriptome assemblies. Node values indicate UFboot supports. Five hymenopteran species were used as an outgroup.

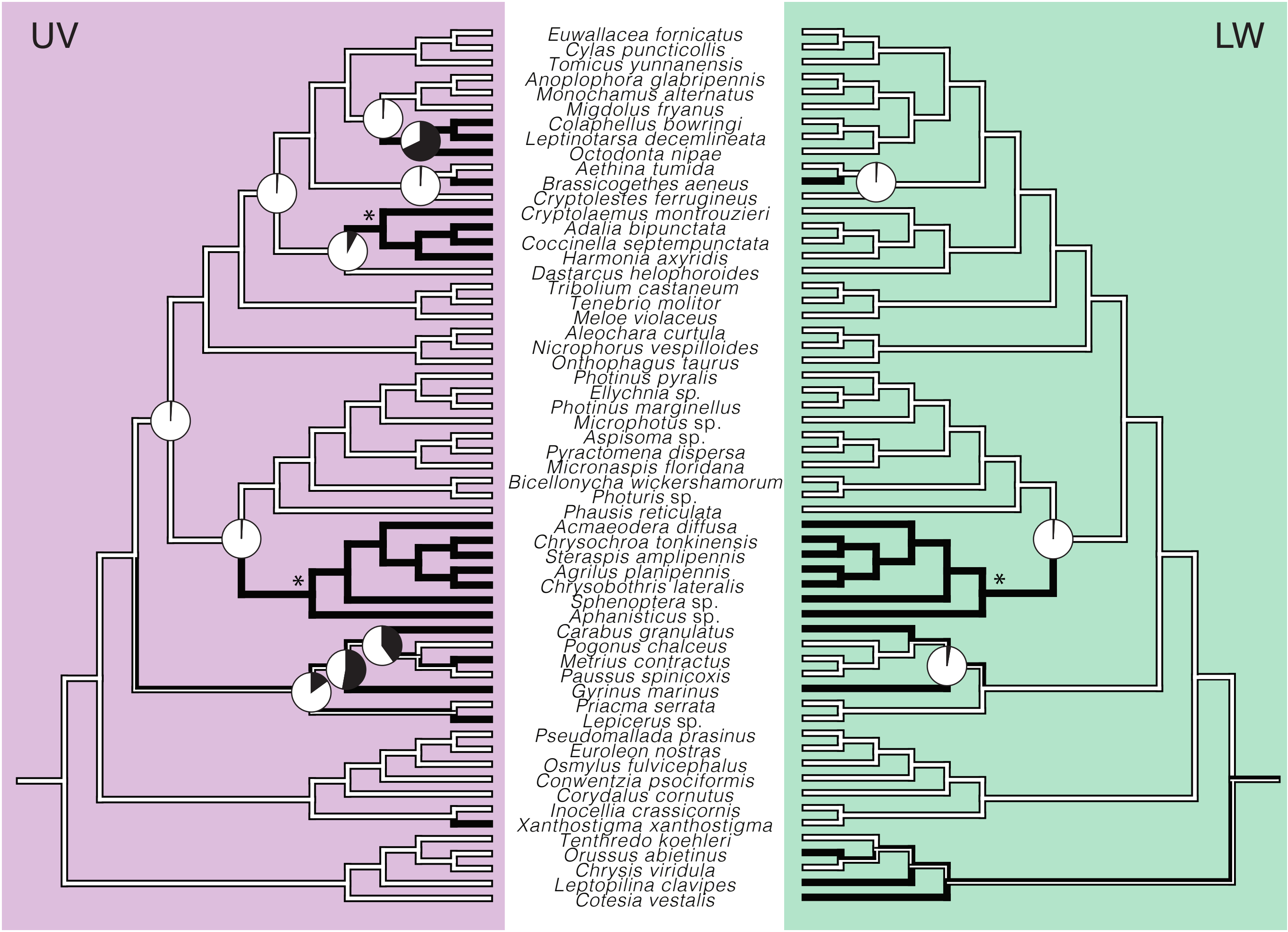

Figure S3. Ancestral state reconstruction for the presence or absence of UV (purple) and LW (green) opsin duplications for all species included in the species phylogeny. The probability that an opsin duplication is present is indicated on the topology (parsimony) and proportionally represented on nodes of interest (likelihood). Black and white indicate the presence or absence of a duplication, respectively. Asterisks denote a likelihood of 1.

Order	Analysis	Species	Family	Superfamily/Suborder	Accession/SRA code	Data type	Gender	Stage (egg - adult)	# animals	Tissue type	Data source	# transcripts	N50	# phototransduction genes	% BUSCOs
Coleoptera	Species phylogeny & opsin phylogeny	*Priacma serrata*	Cupedidae	Archostemata	-	ILLUMINA RNA-seq PE-reads	m	Adult	1	Head	This study	71057	554	15	-
Coleoptera	Opsin phylogeny	*Priacma serrata*	Cupedidae	Archostemata	-	ILLUMINA RNA-seq PE-reads	f	Adult	1	Head	This study	74402	554	13	-
Coleoptera	Opsin phylogeny	*Micromalthus debilis*	Micromalthidae	Archostemata	PRJNA286555	ILLUMINA RNA-seq PE-reads	NA	Larva	10	Whole animal	1KITE unpublished data	28718	1796	11	78
Coleoptera	Opsin phylogeny	*Xylobiops basilaris*	Bostrichidae	Bostrichoidea	PRJNA286597	ILLUMINA RNA-seq PE-reads	NA	Adult	1	Whole animal	1KITE unpublished data	16807	1463	12	75
Coleoptera	Species phylogeny & opsin phylogeny	*Acmaeodera diffusa*	Buprestidae	Buprestoidea	-	ILLUMINA RNA-seq PE-reads	m	Adult	1	Head	(Martin *et al.*, 2015)	142298	414	15	-
Coleoptera	Opsin phylogeny	*Agrilus planipennis*	Buprestidae	Buprestoidea	-	ILLUMINA RNA-seq PE-reads	f	Adult	1	Head	(Lord *et al.*, 2016)	44591	2931	15	-
Coleoptera	Species phylogeny & opsin phylogeny	*Agrilus planipennis*	Buprestidae	Buprestoidea	-	ILLUMINA RNA-seq PE-reads	m	Adult	1	Head	(Martin *et al.*, 2015)	52315	3276	15	-
Coleoptera	Opsin phylogeny	*Agrilus planipennis*	Buprestidae	Buprestoidea	SRR1791270	ILLUMINA RNA-seq PE-reads	m	Larva	NA	Whole animal	Sequence Read Archive	93728	3327	14	89
Coleoptera	Opsin phylogeny	*Aphanisticus* sp.	Buprestidae	Buprestoidea	-	ILLUMINA RNA-seq PE-reads	f	Adult	1	Head	This study	32501	562	13	-
Coleoptera	Species phylogeny & opsin phylogeny	*Aphanisticus* sp.	Buprestidae	Buprestoidea	-	ILLUMINA RNA-seq PE-reads	m	Adult	1	Head	This study	26286	555	10	-
Coleoptera	Species phylogeny & opsin phylogeny	*Chrysobothris lateralis*	Buprestidae	Buprestoidea	-	ILLUMINA RNA-seq PE-reads	m	Adult	1	Head	(Lord *et al.*, 2016)	97466	513	15	-
Coleoptera	Species phylogeny & opsin phylogeny	*Chrysochroa tonkinensis*	Buprestidae	Buprestoidea	-	ILLUMINA RNA-seq PE-reads	m	Adult	1	Head	(Martin *et al.*, 2015)	63672	1789	15	-
Coleoptera	Species phylogeny & opsin phylogeny	*Sphenoptera* sp.	Buprestidae	Buprestoidea	-	ILLUMINA RNA-seq PE-reads	f	Adult	1	Head	(Lord *et al.*, 2016)	68353	478	10	-
Coleoptera	Species phylogeny & opsin phylogeny	*Steraspis amplipennis*	Buprestidae	Buprestoidea	-	ILLUMINA RNA-seq PE-reads	m	Adult	1	Head	(Lord *et al.*, 2016)	49956	661	14	-
Coleoptera	Opsin phylogeny	*Dryops* sp. AD-2015	Dryopidae	Byrrhoidea	PRJNA286519	ILLUMINA RNA-seq PE-reads	NA	Adult	3	Whole animal	1KITE unpublished data	30332	1792	14	83
Coleoptera	Opsin phylogeny	*Anoplophora glabripennis*	Cerambycidae	Chrysomeloidea	GCA_000390285.1	Genome (scaffolds)	-	-	-	-	The i5k Initiative	-	-	-	-
Coleoptera	Species phylogeny	*Anoplophora glabripennis*	Cerambycidae	Chrysomeloidea	SRR1799852	ILLUMINA RNA-seq PE-reads	m	Adult	1	Whole animal	Sequence Read Archive	65411	1987	15	-
Coleoptera	Species phylogeny & opsin phylogeny	*Monochamus alternatus*	Cerambycidae	Chrysomeloidea	SRR907329	ILLUMINA RNA-seq SE-reads	NA	NA	NA	Whole animal	Sequence Read Archive	56640	737	12	73
Coleoptera	Species phylogeny & opsin phylogeny	*Colaphellus bowringi*	Chrysomelidae	Chrysomeloidea	SRR925368	ILLUMINA RNA-seq PE-reads	Both	Larva, pupa and adult	Multiple	Whole animal	Sequence Read Archive	73642	1063	15	88
Coleoptera	Opsin phylogeny	*Leptinotarsa decemlineata*	Chrysomelidae	Chrysomeloidea	SRR1827565	ILLUMINA RNA-seq PE-reads	f	Adult	NA	Whole animal	Sequence Read Archive	103919	1486	15	89
Coleoptera	Species phylogeny & opsin phylogeny	*Leptinotarsa decemlineata*	Chrysomelidae	Chrysomeloidea	SRR1827566	ILLUMINA RNA-seq PE-reads	m	Adult	NA	Whole animal	Sequence Read Archive	83796	1449	15	87
Coleoptera	Species phylogeny & opsin phylogeny	*Octodonta nipae*	Chrysomelidae	Chrysomeloidea	SRR1055951	ILLUMINA RNA-seq PE-reads	NA	All	Multiple	Whole animal	Sequence Read Archive	80606	701	14	74
Coleoptera	Opsin phylogeny	*Oreina cacaliae*	Chrysomelidae	Chrysomeloidea	PRJNA286569	ILLUMINA RNA-seq PE-reads	NA	Adult	1	NA	1KITE unpublished data	40650	1333	11	77
Coleoptera	Species phylogeny & opsin phylogeny	*Migdolus fryanus*	Vesperidae	Chrysomeloidea	SRR1562205	ILLUMINA RNA-seq PE-reads	NA	NA	NA	Whole animal	Sequence Read Archive	61003	1396	11	81
Coleoptera	Opsin phylogeny	*Thanasimus formicarius*	Cleridae	Cleroidea	PRJNA286592	ILLUMINA RNA-seq PE-reads	NA	Adult	1	Whole animal	1KITE unpublished data	34321	1506	11	68
Coleoptera	Species phylogeny	*Adalia bipunctata*	Coccinellidae	Coccinelloidea	ERR1145719	ILLUMINA RNA-seq PE-reads	NA	Adult	Multiple	Whole animal	Sequence Read Archive	36971	1477	12	81
Coleoptera	Opsin phylogeny	*Adalia bipunctata*	Coccinellidae	Coccinelloidea	PRJEB11874	Combined ILLUMINA RNA-seq PE-reads	NA	Adult	Multiple	Whole animal	Sequence Read Archive	69267	1832	15	90
Coleoptera	Species phylogeny	*Coccinella septempunctata*	Coccinellidae	Coccinelloidea	ERR1145724	ILLUMINA RNA-seq PE-reads	NA	Adult	Multiple	Whole animal	Sequence Read Archive	35246	1364	12	78
Coleoptera	Opsin phylogeny	*Coccinella septempunctata*	Coccinellidae	Coccinelloidea	PRJEB11877	Combined ILLUMINA RNA-seq PE-reads	NA	Adult	Multiple	Whole animal	Sequence Read Archive	71218	1656	15	90
Coleoptera	Species phylogeny & opsin phylogeny	*Cryptolaemus montrouzieri*	Coccinellidae	Coccinelloidea	SRR343064	ILLUMINA RNA-seq PE-reads	Both	Adult	Multiple	Whole animal	Sequence Read Archive	46988	811	15	71
Coleoptera	Species phylogeny & opsin phylogeny	*Harmonia axyridis*	Coccinellidae	Coccinelloidea	SRR1283228	ILLUMINA RNA-seq SE-reads	NA	Adult	NA	Whole animal	Sequence Read Archive	101398	652	14	78
Coleoptera	Species phylogeny & opsin phylogeny	*Dastarcus helophoroides*	Bothrideridae	Cucujoidea	SRR1201401	ILLUMINA RNA-seq PE-reads	Both	Adult	4	Whole animal	Sequence Read Archive	67720	728	14	76
Coleoptera	Opsin phylogeny	*Cucujus clavipes*	Cucujidae	Cucujoidea	PRJNA286510	ILLUMINA RNA-seq SE-reads	NA	Adult	1	Whole animal	1KITE unpublished data	20731	1731	13	84
Coleoptera	Species phylogeny & opsin phylogeny	*Cryptolestes ferrugineus*	Laemophloeidae	Cucujoidea	SRR1825376	ILLUMINA RNA-seq SE-reads	Both	Larva, pupa and adult	Multiple	Whole animal	Sequence Read Archive	85044	671	15	70
Coleoptera	Species phylogeny & opsin phylogeny	*Aethina tumida*	Nitidulidae	Cucujoidea	SRR1798556	ILLUMINA RNA-seq PE-reads	f	All	Multiple	Various	Sequence Read Archive	69172	1880	15	-
Coleoptera	Species phylogeny & opsin phylogeny	*Brassicogethes aeneus*	Nitidulidae	Cucujoidea	ERR259383	ILLUMINA RNA-seq SE-reads	NA	Adult	120	Whole animal	Sequence Read Archive	58570	1199	14	86
Coleoptera	Species phylogeny & opsin phylogeny	*Cylas puncticollis*	Curculionidae	Curculionoidea	SRR1611772	ILLUMINA RNA-seq SE-reads	NA	Adult	NA	Whole animal	Sequence Read Archive	86406	2013	15	90
Coleoptera	Opsin phylogeny	*Dendroctonus ponderosae*	Curculionidae	Curculionoidea	GCA_000355655.1	Genome (scaffolds)	-	-	-	-	The Tria Project	-	-	-	-
Coleoptera	Species phylogeny & opsin phylogeny	*Euwallacea fornicatus*	Curculionidae	Curculionoidea	SRR1573502	ILLUMINA RNA-seq PE-reads	f	Adult	NA	Whole animal	Sequence Read Archive	111634	4099	15	90
Coleoptera	Opsin phylogeny	*Hypothenemus hampei*	Curculionidae	Curculionoidea	GCA_001012855.1	Genome (scaffolds)	-	-	-	-	NYU School of Medicine	-	-	-	-
Coleoptera	Opsin phylogeny	*Larinus minutus*	Curculionidae	Curculionoidea	PRJNA286543	ILLUMINA RNA-seq PE-reads	NA	Adult	>2	Whole animal	1KITE unpublished data	29721	1563	12	72
Coleoptera	Species phylogeny & opsin phylogeny	*Tomicus yunnanensis*	Curculionidae	Curculionoidea	DRR013051	ILLUMINA RNA-seq PE-reads	Both	Adult	2	Head	Sequence Read Archive	78011	582	11	-
Coleoptera	Opsin phylogeny	*Anorus arizonicus*	Dascillidae	Dascilloidea	PRJNA286485	ILLUMINA RNA-seq PE-reads	NA	Adult	3	Whole animal	1KITE unpublished data	22370	1906	15	78
Coleoptera	Species phylogeny & opsin phylogeny	*Aspisoma* sp.	Lampyridae	Elateroidea	-	ILLUMINA RNA-seq PE-reads	m	Adult	1	Head	(Martin *et al.*, 2015)	10180	1841	15	-
Coleoptera	Species phylogeny & opsin phylogeny	*Bicellonycha wickershamorum*	Lampyridae	Elateroidea	-	ILLUMINA RNA-seq PE-reads	m	Adult	1	Head	(Martin *et al.*, 2015)	54138	2324	14	-
Coleoptera	Species phylogeny & opsin phylogeny	*Ellychnia* sp.	Lampyridae	Elateroidea	-	ILLUMINA RNA-seq PE-reads	m	Adult	1	Whole animal	(Martin *et al.*, 2015)	56511	2478	15	90
Coleoptera	Species phylogeny & opsin phylogeny	*Micronaspis floridana*	Lampyridae	Elateroidea	-	ILLUMINA RNA-seq PE-reads	m	Adult	1	Head	(Martin *et al.*, 2015)	30157	1468	14	-
Coleoptera	Species phylogeny & opsin phylogeny	*Microphotus* sp.	Lampyridae	Elateroidea	-	ILLUMINA RNA-seq PE-reads	f	Adult	1	Whole animal	(Martin *et al.*, 2015)	63016	2538	12	87
Coleoptera	Species phylogeny & opsin phylogeny	*Phausis reticulata*	Lampyridae	Elateroidea	-	ILLUMINA RNA-seq PE-reads	NA	Adult	1	Whole animal	(Martin *et al.*, 2015)	73905	2837	15	87
Coleoptera	Species phylogeny & opsin phylogeny	*Photinus marginellus*	Lampyridae	Elateroidea	-	ILLUMINA RNA-seq PE-reads	NA	Adult	1	Whole animal	(Martin *et al.*, 2015)	61254	2504	15	90
Coleoptera	Species phylogeny & opsin phylogeny	*Photinus pyralis*	Lampyridae	Elateroidea	-	ILLUMINA RNA-seq PE-reads	m	Adult	1	Whole animal	(Martin *et al.*, 2015)	32337	2005	15	84
Coleoptera	Species phylogeny & opsin phylogeny	*Photuris* sp.	Lampyridae	Elateroidea	-	ILLUMINA RNA-seq PE-reads	m	Adult	1	Head	(Martin *et al.*, 2015)	31346	1817	15	-
Coleoptera	Opsin phylogeny	*Photuris* sp.	Lampyridae	Elateroidea	-	ILLUMINA RNA-seq PE-reads	NA	Larva	1	Whole animal	(Martin *et al.*, 2015)	47007	1462	11	100
Coleoptera	Species phylogeny & opsin phylogeny	*Pyractomena dispersa*	Lampyridae	Elateroidea	-	ILLUMINA RNA-seq PE-reads	m	Adult	1	Head	(Martin *et al.*, 2015)	31768	1071	14	-
Coleoptera	Species phylogeny & opsin phylogeny	*Carabus granulatus*	Carabidae	Geadephaga	SRR596983	ILLUMINA RNA-seq PE-reads	NA	Adult	2	Head and thorax	Sequence Read Archive	112497	989	10	-
Coleoptera	Species phylogeny & opsin phylogeny	*Metrius contractus*	Carabidae	Geadephaga	SRR1727930	ILLUMINA RNA-seq PE-reads	NA	NA	NA	Whole animal	Sequence Read Archive	45779	1065	13	76
Coleoptera	Species phylogeny & opsin phylogeny	*Paussus spinicoxis*	Carabidae	Geadephaga	SRR1727931	ILLUMINA RNA-seq PE-reads	NA	NA	NA	Whole animal	Sequence Read Archive	50843	989	14	77
Coleoptera	Species phylogeny & opsin phylogeny	*Pogonus chalceus*	Carabidae	Geadephaga	SRR424344	ILLUMINA RNA-seq PE-reads	m	Adult	NA	Whole animal	Sequence Read Archive	53358	2327	15	90
Coleoptera	Opsin phylogeny	*Pogonus chalceus*	Carabidae	Geadephaga	SRR424340	ILLUMINA RNA-seq PE-reads	NA	Larva	NA	Whole animal	Sequence Read Archive	41632	2183	13	89
Coleoptera	Opsin phylogeny	*Hypocaccus fitchi*	Histeridae	Histeroidea	PRJNA286537	ILLUMINA RNA-seq PE-reads	NA	Adult	10	Whole animal	1KITE unpublished data	20334	1260	12	75
Coleoptera	Species phylogeny & opsin phylogeny	*Gyrinus marinus*	Gyrinidae	Hydradephaga	SRR921604	ILLUMINA RNA-seq PE-reads	NA	NA	12	Whole animal	Sequence Read Archive	72406	789	13	73
Coleoptera	Opsin phylogeny	*Hydrochara caraboides*	Hydrophilidae	Hydrophiloidea	PRJNA286534	ILLUMINA RNA-seq PE-reads	NA	Larva	1	Whole animal	1KITE unpublished data	25261	1074	10	59
Coleoptera	Species phylogeny & opsin phylogeny	*Lepicerus* sp.	Lepiceridae	Myxophaga	SRR921609	ILLUMINA RNA-seq PE-reads	NA	Adult	16	Whole animal	Sequence Read Archive	198341	563	15	85
Coleoptera	Opsin phylogeny	*Heterochelus* sp. AD-2015	Scarabaeidae	Scarabaeoidea	PRJNA286531	ILLUMINA RNA-seq PE-reads	NA	Adult	1	Whole animal	1KITE unpublished data	50435	965	12	72
Coleoptera	Opsin phylogeny	*Onthophagus taurus*	Scarabaeidae	Scarabaeoidea	SRR1864888	ILLUMINA RNA-seq PE-reads	f	Adult	NA	Whole animal	Sequence Read Archive	104655	1899	15	90
Coleoptera	Species phylogeny & opsin phylogeny	*Onthophagus taurus*	Scarabaeidae	Scarabaeoidea	SRR1864900	ILLUMINA RNA-seq PE-reads	m	Adult	NA	Whole animal	Sequence Read Archive	128111	1676	15	90
Coleoptera	Opsin phylogeny	*Onthophagus taurus*	Scarabaeidae	Scarabaeoidea	SRR1864877	ILLUMINA RNA-seq PE-reads	m	Pupa	NA	Whole animal	Sequence Read Archive	108453	1831	15	90
Coleoptera	Opsin phylogeny	*Nicrophorus vespilloides*	Silphidae	Staphylinoidea	SRR2089961	ILLUMINA RNA-seq PE-reads	f	Adult	NA	Head	Sequence Read Archive	43845	1566	15	-
Coleoptera	Species phylogeny & opsin phylogeny	*Nicrophorus vespilloides*	Silphidae	Staphylinoidea	SRR2089993	ILLUMINA RNA-seq PE-reads	m	Adult	NA	Head	Sequence Read Archive	40076	1409	14	-
Coleoptera	Species phylogeny & opsin phylogeny	*Aleochara curtula*	Staphylinidae	Staphylinoidea	SRR921563	ILLUMINA RNA-seq PE-reads	Both	Adult	10	Whole animal	Sequence Read Archive	43803	1621	11	82
Coleoptera	Species phylogeny & opsin phylogeny	*Meloe violaceus*	Meloidae	Tenebrionoidea	SRR921618	ILLUMINA RNA-seq PE-reads	NA	Adult	2	Whole animal	Sequence Read Archive	46893	1121	15	83
Coleoptera	Opsin phylogeny	*Macrosiagon limbatum*	Ripiphoridae	Tenebrionoidea	PRJNA286549	ILLUMINA RNA-seq PE-reads	f	Adult	1	Whole animal	1KITE unpublished data	26483	1096	13	69
Coleoptera	Opsin phylogeny	*Lagria hirta*	Tenebrionidae	Tenebrionoidea	PRJNA286540	ILLUMINA RNA-seq PE-reads	NA	Adult	3	Whole animal	1KITE unpublished data	40362	1421	15	85
Coleoptera	Species phylogeny & opsin phylogeny	*Tenebrio molitor*	Tenebrionidae	Tenebrionoidea	SRR1291244	ILLUMINA RNA-seq PE-reads	NA	Larva, pupa and adult	Multiple	Whole animal	Sequence Read Archive	85822	1979	15	88
Coleoptera	Opsin phylogeny	*Tribolium castaneum*	Tenebrionidae	Tenebrionoidea	GCA_000002335.3	Genome (chromosomes)	-	-	-	-	Baylor College of Medicine	-	-	-	-
Coleoptera	Opsin phylogeny	*Tribolium castaneum*	Tenebrionidae	Tenebrionoidea	SRR1647933	ILLUMINA RNA-seq PE-reads	f	Adult	NA	Whole animal	Sequence Read Archive	64309	3029	15	89
Coleoptera	Species phylogeny & opsin phylogeny	*Tribolium castaneum*	Tenebrionidae	Tenebrionoidea	SRR1647936	ILLUMINA RNA-seq PE-reads	m	Adult	NA	Whole animal	Sequence Read Archive	43732	2868	15	90
Coleoptera	Opsin phylogeny	*Tribolium castaneum*	Tenebrionidae	Tenebrionoidea	SRR1647930	ILLUMINA RNA-seq PE-reads	Both	Larva	NA	Whole animal	Sequence Read Archive	34860	2584	15	90
Hymenoptera	Species phylogeny & opsin phylogeny	*Chrysis viridula*	Chrysididae	Chrysidoidea	SRR921581	ILLUMINA RNA-seq PE-reads	Both	Adult	7	Whole animal	Sequence Read Archive	64858	1727	14	84
Hymenoptera	Species phylogeny & opsin phylogeny	*Leptopilina clavipes*	Figitidae	Cynipoidea	SRR921610	ILLUMINA RNA-seq PE-reads	Both	Pupa and adult	194	Whole animal	Sequence Read Archive	42426	1411	13	87
Hymenoptera	Species phylogeny & opsin phylogeny	*Cotesia vestalis*	Braconidae	Ichneumonoidea	SRR921586	ILLUMINA RNA-seq PE-reads	NA	Larva and adult	40	Whole animal	Sequence Read Archive	56770	1019	14	86
Hymenoptera	Species phylogeny & opsin phylogeny	*Orussus abietinus*	Orussidae	Orussoidea	SRR921626	ILLUMINA RNA-seq PE-reads	Both	Adult	3	Whole animal	Sequence Read Archive	69615	2747	14	87
Hymenoptera	Species phylogeny & opsin phylogeny	*Tenthredo koehleri*	Tenthredinidae	Tenthredinoidea	SRR921645	ILLUMINA RNA-seq PE-reads	Both	Adult	4	Whole animal	Sequence Read Archive	57564	2066	12	89
Megaloptera	Species phylogeny & opsin phylogeny	*Corydalus cornutus*	Corydalidae	-	SRR921584	ILLUMINA RNA-seq PE-reads	NA	NA	1	Whole animal	Sequence Read Archive	36512	1515	12	83
Neuroptera	Species phylogeny & opsin phylogeny	*Osmylus fulvicephalus*	Osmylidae	-	SRR921627	ILLUMINA RNA-seq PE-reads	NA	Adult	2	Whole animal	Sequence Read Archive	49206	1601	14	83
Neuroptera	Species phylogeny & opsin phylogeny	*Pseudomallada prasinus*	Chrysopidae	Chrysopoidea	SRR921589	ILLUMINA RNA-seq PE-reads	NA	Adult	3	Whole animal	Sequence Read Archive	153592	712	13	87
Neuroptera	Species phylogeny & opsin phylogeny	*Conwentzia psociformis*	Coniopterygidae	Coniopterygoidea	SRR921582	ILLUMINA RNA-seq PE-reads	NA	Adult	8	Whole animal	Sequence Read Archive	90140	1054	15	85
Neuroptera	Species phylogeny & opsin phylogeny	*Euroleon nostras*	Myrmeleontidae	Myrmeleontoidea	SRR921595	ILLUMINA RNA-seq PE-reads	NA	Adult	1	Whole animal	Sequence Read Archive	81830	603	14	77
Raphidioptera	Species phylogeny & opsin phylogeny	*Xanthostigma xanthostigma*	Raphidiidae	-	SRR921657	ILLUMINA RNA-seq PE-reads	f	Adult	1	Whole animal	Sequence Read Archive	126312	718	13	82
Raphidioptera	Species phylogeny & opsin phylogeny	*Inocellia crassicornis*	Inocelliidae	Raphidiomorpha	SRR921607	ILLUMINA RNA-seq PE-reads	NA	Larva	6	Whole animal	Sequence Read Archive	81878	827	13	78
Strepsiptera	Species phylogeny & opsin phylogeny	*Mengenilla moldrzyki*	Mengenillidae	-	SRR619393	ILLUMINA RNA-seq PE-reads	m	Adult	NA	Whole animal	Sequence Read Archive	231777	684	11	38
Strepsiptera	Species phylogeny & opsin phylogeny	*Stylops melittae*	Stylopidae	-	SRR921643	ILLUMINA RNA-seq PE-reads	m	Adult	8	Whole animal	Sequence Read Archive	55336	3161	15	72
Strepsiptera	Opsin phylogeny	*Xenos vesparum*	Xenidae	-	SRR1784897	ILLUMINA RNA-seq PE-reads	f	Adult (neotenic)	NA	Whole animal	Sequence Read Archive	116890	665	13	67
Strepsiptera	Opsin phylogeny	*Xenos vesparum*	Xenidae	-	SRR1784898	ILLUMINA RNA-seq PE-reads	f	Larva	NA	Whole animal	Sequence Read Archive	102600	828	15	71
Strepsiptera	Species phylogeny & opsin phylogeny	*Xenos vesparum*	Xenidae	-	SRR1784886	ILLUMINA RNA-seq PE-reads	m	Pupa	NA	Whole animal	Sequence Read Archive	123082	720	15	70

Table S1. Sample information for all sequence data used in this study. Sample information that is not available is denoted by NA. Later versions of 1KITE assemblies were used (version e3; see www.1kite.org). Percent complete BUSCOs are only reported for whole body transcriptomes.

Order	Species	Family	Superfamily/Suborder	UV	SW	LW
Coleoptera	*Carabus granulatus*	Carabidae	Adephaga	2	0	2
Coleoptera	*Metrius contractus*	Carabidae	Adephaga	2*	0	1
Coleoptera	*Paussus spinicoxis*	Carabidae	Adephaga	1	0	1
Coleoptera	*Pogonus chalceus*	Carabidae	Adephaga	1	0	1
Coleoptera	*Gyrinus marinus*	Gyrinidae	Adephaga	2	0	2
Coleoptera	*Micromalthus debilis*	Micromalthidae	Archostemata	1	0	1
Coleoptera	*Thanasimus formicarius*	Cleridae	Cleroidea	2	0	1
Coleoptera	*Xylobiops basilaris*	Bostrichidae	Bostrichoidea	1	0	1
Coleoptera	*Acmaeodera diffusa*	Buprestidae	Buprestoidea	2	0	2
Coleoptera	*Agrilus planipennis*	Buprestidae	Buprestoidea	2	0	2
Coleoptera	*Aphanisticus* sp.	Buprestidae	Buprestoidea	2	0	2
Coleoptera	*Chrysobothris lateralis*	Buprestidae	Buprestoidea	2	0	2
Coleoptera	*Chrysochroa tonkinensis*	Buprestidae	Buprestoidea	2	0	2
Coleoptera	*Sphenoptera* sp.	Buprestidae	Buprestoidea	2	0	2
Coleoptera	*Steraspis amplipennis*	Buprestidae	Buprestoidea	2	0	2
Coleoptera	*Dryops* sp. AD-2015	Dryopidae	Byrrhoidea	2	0	1
Coleoptera	*Anoplophora glabripennis*	Cerambycidae	Chrysomeloidea	1	0	1
Coleoptera	*Monochamus alternatus*	Cerambycidae	Chrysomeloidea	0	0	1
Coleoptera	*Colaphellus bowringi*	Chrysomelidae	Chrysomeloidea	2	0	1
Coleoptera	*Leptinotarsa decemlineata*	Chrysomelidae	Chrysomeloidea	2	0	1
Coleoptera	*Octodonta nipae*	Chrysomelidae	Chrysomeloidea	2	0	1
Coleoptera	*Oreina cacaliae*	Chrysomelidae	Chrysomeloidea	3*	0	1
Coleoptera	*Migdolus fryanus*	Vesperidae	Chrysomeloidea	2	0	1
Coleoptera	*Cryptolaemus montrouzieri*	Coccinellidae	Coccinelloidea	2	0	1
Coleoptera	*Dastarcus helophoroides*	Bothrideridae	Cucujoidea	0	0	1
Coleoptera	*Adalia bipunctata*	Coccinellidae	Cucujoidea	2	0	1
Coleoptera	*Coccinella septempunctata*	Coccinellidae	Cucujoidea	2	0	1
Coleoptera	*Harmonia axyridis*	Coccinellidae	Cucujoidea	2	0	1
Coleoptera	*Cryptolestes ferrugineus*	Laemophloeidae	Cucujoidea	1	0	1
Coleoptera	*Aethina tumida*	Nitidulidae	Cucujoidea	1	0	1
Coleoptera	*Brassicogethes aeneus*	Nitidulidae	Cucujoidea	3	0	2
Coleoptera	*Priacma serrata*	Cupedidae	Archostemata	1	0	1
Coleoptera	*Cylas puncticollis*	Curculionidae	Curculionoidea	1	0	1
Coleoptera	*Dendroctonus ponderosae*	Curculionidae	Curculionoidea	1	0	1
Coleoptera	*Hypothenemus hampei*	Curculionidae	Curculionoidea	1	0	1
Coleoptera	*Larinus minutus*	Curculionidae	Curculionoidea	1	0	3
Coleoptera	*Euwallacea fornicatus*	Scolytinae	Curculionoidea	1	0	1
Coleoptera	*Tomicus yunnanensis*	Scolytinae	Curculionoidea	2*	0	1
Coleoptera	*Anorus arizonicus*	Dascillidae	Dascilloidea	1	0	1
Coleoptera	*Aspisoma* sp.	Lampyridae	Elateroidea	1	0	1
Coleoptera	*Bicellonycha wickershamorum*	Lampyridae	Elateroidea	1	0	1
Coleoptera	*Ellychnia* sp.	Lampyridae	Elateroidea	1	0	1
Coleoptera	*Micronaspis floridana*	Lampyridae	Elateroidea	1	0	1
Coleoptera	*Microphotus* sp.	Lampyridae	Elateroidea	1	0	0
Coleoptera	*Phausis reticulata*	Lampyridae	Elateroidea	1	0	1
Coleoptera	*Photinus marginellus*	Lampyridae	Elateroidea	1	0	1
Coleoptera	*Photinus pyralis*	Lampyridae	Elateroidea	1	0	1
Coleoptera	*Photuris* sp.	Lampyridae	Elateroidea	1	0	1
Coleoptera	*Pyractomena dispersa*	Lampyridae	Elateroidea	1	0	1
Coleoptera	*Hydrochara caraboides*	Hydrophilidae	Hydrophiloidea	1	0	1
Coleoptera	*Lepicerus* sp.	Lepiceridae	Myxophaga	2	0	1
Coleoptera	*Cucujus clavipes*	Cucujidae	Cucujoidea	1	0	1
Coleoptera	*Heterochelus* sp. AD-2015	Scarabaeidae	Scarabaeoidea	1	0	2
Coleoptera	*Onthophagus taurus*	Scarabaeidae	Scarabaeoidea	1	0	1
Coleoptera	*Nicrophorus vespilloides*	Silphidae	Staphylinoidea	1	0	1
Coleoptera	*Aleochara curtula*	Staphylinidae	Staphylinoidea	1	0	1
Coleoptera	*Meloe violaceus*	Meloidae	Tenebrionoidea	1	0	1
Coleoptera	*Macrosiagon limbatum*	Ripiphoridae	Tenebrionoidea	1	0	1
Coleoptera	*Hypocaccus fitchi*	Histeridae	Histeroidea	1	0	1
Coleoptera	*Lagria hirta*	Tenebrionidae	Tenebrionoidea	1	0	1
Coleoptera	*Tenebrio molitor*	Tenebrionidae	Tenebrionoidea	0	0	1
Coleoptera	*Tribolium castaneum*	Tenebrionidae	Tenebrionoidea	1	0	1
Strepsiptera	*Mengenilla moldrzyki*	Mengenillidae	-	1	0	2
Strepsiptera	*Stylops melittae*	Stylopidae	-	1	0	1
Strepsiptera	*Xenos vesparum*	Xenidae	-	2	0	6
Raphidioptera	*Xanthostigma xanthostigma*	Raphidiidae	-	2	0	1
Raphidioptera	*Inocellia crassicornis*	Inocelliidae	Raphidiomorpha	1	0	1
Megaloptera	*Corydalus cornutus*	Corydalidae	-	1	0	1
Neuroptera	*Osmylus fulvicephalus*	Osmylidae	-	1	0	1
Neuroptera	*Pseudomallada prasinus*	Chrysopidae	Chrysopoidea	1	0	1
Neuroptera	*Conwentzia psociformis*	Coniopterygidae	Coniopterygoidea	1	0	1
Neuroptera	*Euroleon nostras*	Myrmeleontidae	Myrmeleontoidea	1	0	1
Hymenoptera	*Chrysis viridula*	Chrysididae	Chrysidoidea	1	1	1
Hymenoptera	*Leptopilina clavipes*	Figitidae	Cynipoidea	1	1	2
Hymenoptera	*Cotesia vestalis*	Braconidae	Ichneumonoidea	1	1	2
Hymenoptera	*Orussus abietinus*	Orussidae	Orussoidea	1	1	2
Hymenoptera	*Tenthredo koehleri*	Tenthredinidae	Tenthredinoidea	1	1	1

Table S2. Opsin copy number for all species sampled in this study. Asterisks indicate two opsin duplicates that do not overlap and therefore may represent a single opsin copy.

Table S3. Lengths of Neuropteroidea opsin protein sequences found in this study. Sequence identity and coverage is compared for opsin duplicates. Opsin sequences derived from RNA-seq data can be found on GenBank with accession numbers KY368182 - KY368379.

**Opsin sequence**	**length (AAs)**	**Sequence identity (AA) (%)**	**Sequence identity (nucleotide) (%)**	**Sequence coverage (AA) (%)**
Acmaeodera diffusa LW1	379	61	66	94
Acmaeodera diffusa LW2	378			
Acmaeodera diffusa UV1	377	63	65	91
Acmaeodera diffusa UV2	390			
Adalia bipunctata LW	375			
Adalia bipunctata UV1	391	84	77	96
Adalia bipunctata UV2	389			
Aethina tumida LW	380			
Aethina tumida UV	382			
Agrilus planipennis LW1	379	69	70	98
Agrilus planipennis LW2	380			
Agrilus planipennis LW3	327			
Agrilus planipennis UV1	372	72	69	91
Agrilus planipennis UV2	394			
Aleochara curtula LW	381			
Aleochara curtula UV	380			
Anoplophora glabripennis LW	377			
Anoplophora glabripennis UV	319			
Anorus arizonicus LW	378			
Anorus arizonicus UV	386			
Aphanisticus sp. LW1	269	69	67	74
Aphanisticus sp. LW2	381			
Aphanisticus sp. UV1	377	72	70	96
Aphanisticus sp. UV2	375			
Aspisoma sp. LW	380			
Aspisoma sp. UV	397			
Bicellonycha wickershamorum LW	380			
Bicellonycha wickershamorum UV	385			
Brassicogethes aeneus LW1	398	78	74	94
Brassicogethes aeneus LW2	381			
Brassicogethes aeneus UV1	380	82	74	79
Brassicogethes aeneus UV3	278			
Brassicogethes aeneus UV2	382			
Carabus granulatus LW1	364	100	99	53
Carabus granulatus LW2	137			
Carabus granulatus UV1	123	87	74	32
Carabus granulatus UV2	381			
Chrysobothris lateralis LW1	381	69	69	99
Chrysobothris lateralis LW2	380			
Chrysobothris lateralis UV1	375	68	70	97
Chrysobothris lateralis UV2	378			
Chrysochroa tonkinensis LW1	381	68	71	99
Chrysochroa tonkinensis LW2	380			
Chrysochroa tonkinensis UV1	385	74	70	96
Chrysochroa tonkinensis UV2	371			
Coccinella septempunctata LW	375			
Coccinella septempunctata UV1	396	83	78	98
Coccinella septempunctata UV2	389			
Colaphellus bowringi LW	375			
Colaphellus bowringi UV1	360	77	75	58
Colaphellus bowringi UV2	181			
Conwentzia psociformis LW	371			
Conwentzia psociformis UV	380			
Corydalus cornutus LW	376			
Corydalus cornutus UV	347			
Cryptolaemus montrouzieri LW	376			
Cryptolaemus montrouzieri UV1a	398	86	79	95
Cryptolaemus montrouzieri UV1b	376			
Cryptolestes ferrugineus LW	378			
Cryptolestes ferrugineus UV	236			
Cucujus clavipes LW	378			
Cucujus clavipes UV	391			
Cylas puncticollis LW	379			
Cylas puncticollis UV	374			
Dastarcus helophoroides LW	378			
Dendroctonus ponderosae LW	379			
Dendroctonus ponderosae UV	368			
Dryops sp. AD-2015 LW	382			
Dryops sp. AD-2015 UV1	181	81	75	55
Dryops sp. AD-2015 UV2	370			
Ellychnia sp. LW	379			
Ellychnia sp. UV	389			
Euroleon nostras LW	371			
Euroleon nostras UV	376			
Euwallacea fornicatus LW	378			
Euwallacea fornicatus UV	375			
Gyrinus marinus LW1	379	93	90	36
Gyrinus marinus LW2	104			
Gyrinus marinus UV1	387	89	85	73
Gyrinus marinus UV2	276			
Harmonia axyridis LW	361			
Harmonia axyridis UV1	393	81	75	73
Harmonia axyridis UV2	371			
Heterochelus sp. AD-2015 LW1	151	81	91	44
Heterochelus sp. AD-2015 LW2	376			
Heterochelus sp. AD-2015 UV	213			
Hydrochara caraboides LW	385			
Hydrochara caraboides UV	323			
Hypocaccus fitchi LW	381			
Hypocaccus fitchi UV	180			
Hypothenemus hampei LW	317			
Hypothenemus hampei UV	323			
Inocellia crassicornis LW	378			
Inocellia crassicornis UV	109			
Largria hirta LW	410			
Largria hirta UV	379			
Larinus minutus LW1	219	79	87	71
Larinus minutus LW3	379			
Larinus minutus LW2	149			
Larinus minutus UV	376			
Lepicerus sp. LW	375			
Lepicerus sp. UV1	188	95	88	49
Lepicerus sp. UV2	389			
Leptinotarsa decemlineata LW	410			
Leptinotarsa decemlineata UV1	379	74	73	96
Leptinotarsa decemlineata UV2	389			
Macrosiagon limbatum LW	377			
Macrosiagon limbatum UV	378			
Meloe violaceus LW	380			
Meloe violaceus UV	327			
Mengenilla moldrzyki LW1	243	66	68	66
Mengenilla moldrzyki LW2	383			
Mengenilla moldrzyki UV	135			
Metrius contractus LW	379			
Metrius contractus UV1	197	-	-	0
Metrius contractus UV2	138			
Micromalthus debilis LW	134			
Micromalthus debilis UV	379			
Micronaspis floridana LW	380			
Micronaspis floridana UV	394			
Microphotus sp. UV	389			
Migdolus fryanus LW	360			
Monochamus alternatus LW	378			
Nicrophorus vespilloides LW	380			
Nicrophorus vespilloides UV	388			
Octodonta nipae LW	380			
Octodonta nipae UV1	379	65	71	98
Octodonta nipae UV2	380			
Onthophagus taurus LW	393			
Onthophagus taurus UV	376			
Oreina cacaliae LW	377			
Oreina cacaliae UV1a	126	66	71	96
Oreina cacaliae UV2	184			
Oreina cacaliae UV1b	161			
Osmylus fulvicephalus LW	372			
Osmylus fulvicephalus UV	256			
Paussus spinicoxis LW	381			
Paussus spinicoxis UV	385			
Phausis reticulata LW	413			
Phausis reticulata UV	404			
Photinus marginellus LW	379			
Photinus marginellus UV	401			
Photinus pyralis LW	379			
Photinus pyralis UV	392			
Photuris sp. LW	381			
Photuris sp. UV	386			
Pogonus chalceus LW	383			
Pogonus chalceus UV	386			
Priacma serrata LW	378			
Priacma serrata UV	376			
Pseudomallada prasinus LW	372			
Pseudomallada prasinus UV	395			
Pyractomena dispersa LW	380			
Pyractomena dispersa UV	402			
Sphenoptera sp. LW1	386	71	70	96
Sphenoptera sp. LW2	380			
Sphenoptera sp. UV1	381	73	70	96
Sphenoptera sp. UV2	373			
Steraspis amplipennis LW1	381	70	70	99
Steraspis amplipennis LW2	380			
Steraspis amplipennis UV1	379	72	69	92
Steraspis amplipennis UV2	375			
Stylops melittae LW	237			
Stylops melittae UV	376			
Tenebrio molitor LW	386			
Thanasimus formicarius LW	359			
Thanasimus formicarius UV1	212	87	91	36
Thanasimus formicarius UV2	155			
Tomicus yunnanensis LW	379			
Tomicus yunnanensis UV	313			
Tribolium castaneum LW	379			
Tribolium castaneum UV	382			
Xanthostigma xanthostigma LW1	375	70	69	96
Xanthostigma xanthostigma LW2	377			
Xanthostigma xanthostigma UV1	381	87	82	95
Xanthostigma xanthostigma UV2	394			
Xenos vesparum LW1	369			
Xenos vesparum LW2	269			
Xenos vesparum LW3	208			
Xenos vesparum LW4	245			
Xenos vesparum LW5	124			
Xenos vesparum LW6	328			
Xenos vesparum UV1	374	96	99	62
Xenos vesparum UV2	182			
Xylobiops basilaris LW	385			
Xylobiops basilaris UV	381			

Clade	Family	Branch	Branch leading to	Site numbers (bovine)	Branch P-value	Test lnL	Null lnL	LRT
**A**	Chrysomelidae	A1	UV2	90†, 91*	0.002	-189798.8	-189803.7	9.8
**A**	Chrysomelidae	A2	UV2	29*, 107**,197***, **210****, 230***, 339*	0.000	-189796.8	-189806.7	19.7
**A**	Chrysomelidae	A3	UV2	136*, 151*	0.004	-189803.7	-189807.8	8.2
**A**	Chrysomelidae	A4	UV1	33**, 107*, 127*, 286***, **293*****	0.000	-189797.1	-189803.7	13.2
**A**	Chrysomelidae	A5	UV1	205*	0.021	-189803.6	-189806.3	5.3
**B**	Coccinellidae	B1	UV1/VU2	14***, 31***, 34***, 158*, 159**, 173**, 317*, 333***, 341*	0.000	-189810.2	-189835.0	49.8
**C**	Buprestidae	C1	UV2	96*	0.046	-189803.4	-189805.4	4.0
**C**	Buprestidae	C2	UV2	37*, 105**, 108**, **112***, 164***	0.000	-189806.9	-189819.4	24.9
**C**	Buprestidae	C3	UV1	50*, 127*, **206*****	0.000	-189814.8	-189821.2	12.7
**C**	Buprestidae	C4	UV1	22**, 23*	0.031	-189801.4	-189803.7	4.6
**C**	Buprestidae	C5	UV1	176*, 203*, 258*	0.000	-189797.3	-189803.7	12.9

Table S4. Branches and amino acid sites under positive selection in clades A (Chrysomelidae), B (Coccinellidae) and C (Buprestidae). Sites adjacent to chromophore binding sites are in bold. LRT = Likelihood Ratio Test, lnL = log likelihood. Sites are numbered according to bovine rhodopsin. *** $P \leq 0.001$ ** $P \leq 0.01$, * $P \leq 0.05$. † Site 90 is included as it is close to significance ($P = 0.055$) and is an important site for shifts between UV and blue sensitivity in insect photopigments.

Clade A Chrysomelidae																		
Branch	**A2**	**A4**	**A1**	**A1**	**A2**	**-**	**A4**	**A3**	**A3**	**A4**	**A4**	**A2**	**A5**	**A2**	**A2**	**A4**	**A4**	**A2**
UV clade	UV2	UV1	UV2	UV2	UV2	-	UV1	UV2	UV2	UV1	UV1	UV2	UV1	UV2	UV2	UV2	UV1	UV2
AA site (bovine)	29	33	90	91	107	113	127	136	151	107	172	197	205	210	230	286	293	339
**_Colaphellus bowringi_ UV1**	V	I	K	S	H	F	T	Y	T	M	A	T	G	F	F	T	C	S
**_Leptinotarsa decemlineata_ UV1**	L	V	K	S	Y	F	A	Y	T	M	A	T	A	F	F	T	C	S
**_Octodonta nipae_ UV1**	A	P	K	S	I	F	L	F	T	T	L	T	G	F	F	G	I	N
**_Oreina cacaliae_ UV1a**	-	-	K	S	H	F	T	Y	T	M	T	-	-	-	-	-	-	-
**_Oreina cacaliae_ UV1b**	-	-	-	-	-	-	-	-	-	-	-	-	A	F	F	T	C	S
**_Colaphellus bowringi_ UV2**	-	-	-	-	W	Y	M	Y	T	A	M	D	G	T	F	-	-	-
**_Leptinotarsa decemlineata_ UV2**	Q	P	V	M	W	Y	M	F	P	A	M	D	G	M	F	G	L	L
**_Octodonta nipae_ UV2**	I	P	S	I	I	Y	M	Y	T	A	M	T	G	C	I	G	L	T
**_Oreina cacaliae_ UV2**	Q	P	V	M	W	Y	M	F	P	A	F	-	-	-	-	-	-	-

Clade B Coccinelidae											
Branch	**B1**	**B1**	**B1**	**-**	**-**	**B1**	**B1**	**B1**	**B1**	**B1**	**B1**
UV clade	UV1/2	UV1/2	UV1/2	UV1/2	UV1/2	UV1/2	UV1/2	UV1/2	UV1/2	UV1/2	UV1/2
AA site (bovine)	14	31	34	90	113	158	159	173	317	333	341
**_Adalia bipunctata_ UV1**	S	L	P	K	F	L	V	T	Q	V	E
**_Coccinella septempunctata_ UV1**	S	L	P	K	F	L	L	T	Q	V	E
**_Cryptolaemus montrouzieri_ UV1**	S	T	P	K	F	V	A	T	Q	T	Q
**_Harmonia axyridis_ UV1**	S	S	P	K	F	L	I	T	Q	V	E
**_Adalia bipunctata_ UV2**	S	M	P	K	Y	L	G	T	Q	V	-
**_Coccinella septempunctata_ UV2**	S	I	P	K	F	V	G	L	Q	V	-
**_Cryptolaemus montrouzieri_ UV2**	S	M	P	K	F	L	G	T	L	V	-
**_Harmonia axyridis_ UV2**	S	T	P	S	Y	L	G	I	Q	L	-

Clade C Buprestidae																
Branch	**C1**	**C4**	**C4**	**C2**	**C3**	**-**	**C2**	**C2**	**C2**	**-**	**C3**	**C2**	**C5**	**C3**	**C5**	**C5**
UV clade	UV2	UV1	UV1	UV2	UV1	-	UV2	UV2	UV2	-	UV1	UV2	UV1	UV1	UV1	UV1
AA site (bovine)	14	22	23	37	50	90	105	108	112	113	127	164	176	206	230	258
**_Acmaeodera diffusa_ UV1**	G	P	P	L	L	K	A	L	I	F	M	T	N	V	R	T
**_Agrilus planipennis_ UV1**	A	P	T	L	L	K	T	L	I	F	I	T	N	A	R	T
**_Aphanisticus_ sp. UV1**	S	P	P	L	L	K	T	I	I	F	I	T	N	A	Q	T
**_Chrysobothris lateralis_ UV1**	A	P	P	L	M	K	T	I	I	F	V	T	N	A	R	T
**_Chrysochroa tonkinensis_ UV1**	A	D	N	I	M	K	T	I	I	F	T	T	S	A	H	T
**_Sphenoptera_ sp. UV1**	A	P	P	L	M	K	T	I	I	F	A	T	N	A	R	T
**_Steraspis amplipennis_ UV1**	A	D	S	I	M	E	T	I	I	F	A	T	G	A	H	T
**_Acmaeodera diffusa_ UV2**	S	S	P	L	M	K	L	V	V	F	M	V	G	T	D	C
**_Agrilus planipennis_ UV2**	A	P	A	I	M	K	L	T	A	F	M	V	G	A	D	C
**_Aphanisticus_ sp. UV2**	P	P	A	I	M	K	L	T	T	F	M	V	G	A	N	C
**_Chrysobothris lateralis_ UV2**	Q	P	A	W	M	K	T	L	I	F	M	T	G	T	A	C
**_Chrysochroa tonkinensis_ UV2**	A	P	A	L	M	K	G	V	I	F	M	T	G	G	D	C
**_Sphenoptera_ sp. UV2**	R	P	A	L	M	K	T	L	I	F	M	T	G	S	S	C
**_Steraspis amplipennis_ UV2**	A	P	A	L	M	K	T	L	I	F	M	T	G	A	D	C

Table S5. Amino acids present at sites under positive selection, for UV1 and UV2 opsins in clades A (Chrysomelidae), B (Cocclinellidae), and C (Buprestidae). Sites are numbered according to bovine rhodopsin. Sites 90 and 113 are included as they differ between UV1 and UV2 opsins but are invariant in other insect UV opsins.

Species	BioProject ID	Taxonomy ID	TSA accession #	Version
Anorus arizonicus	PRJNA286485	1661808	GDLJ00000000	GDLJ01000000
Cucujus clavipes	PRJNA286510	186067	GDNW00000000	GDNW01000000
Dryops sp. AD-2015	PRJNA286519	1661493	GDMY00000000	GDMY01000000
Heterochelus sp. AD-2015	PRJNA286531	1661495	GDNJ00000000	GDNJ01000000
Hydrochara caraboides	PRJNA286534	1208885	GDPR00000000	GDPR01000000
Hypocaccus fitchi	PRJNA286537	1661832	GDLI00000000	GDPQ01000000
Lagria hirta	PRJNA286540	296003	GDPQ00000000	GDLA01000000
Larinus minutus	PRJNA286543	1342029	GDLA00000000	GDPU01000000
Macrosiagon limbatum	PRJNA286549	1661804	GDPU00000000	GDOQ01000000
Micromalthus debilis	PRJNA286555	292448	GDOQ00000000	GDPL01000000
Oreina cacaliae	PRJNA286569	63706	GDPL00000000	GDPC01000000
Thanasimus formicarius	PRJNA286592	295689	GDPC00000000	GDLI01000000
Xylobiops basilaris	PRJNA286597	1661920	GDNM00000000	GDNM01000000

Table S6. 1KITE sample information (see www.1kite.org). All samples are derived from one project (accession PRJNA183205).

Table S7. Accession numbers for insect and cephalopod (outgroup) opsin sequences included in phylogenetic and selection analyses.

Opsin sequence	Accession #	Opsin sequence	Accession #	Opsin sequence	Accession #
Allodessus bistrigatus UV	KP219384	*Heliconius melpomene* LW	GU324702	*Nilaparvata lugens* LW	AB761147
Allodessus bistrigatus LW	KP219381	*Heliconius erato* SW	AY918906	*Nilaparvata lugens* UV1	AB761148
Limbodessus palmulaoides LW	KP219382	*Heliconius erato* LW	AY918907	*Nilaparvata lugens* UV2	AB761149
Paroster nigroadumbratus UV	KP219383	*Heliconius erato* UV1	AY918904	*Pieris rapae* blue	AB208675
Paroster nigroadumbratus LW	KP219380	*Heliconius erato* UV2	AY918905	*Pieris rapae* LW	AB177984
Apis cerana SW	AB355817	*Heliconius sapho* SW	GU324692	*Pieris rapae* UV	AB208673
Apis cerana LW	AB355818	*Heliconius sapho* LW	GU324705	*Pieris rapae* violet	AB208674
Apis cerana UV	AB355816	*Heliconius sapho* UV1	GQ451907	*Sogatella furcifera* LW	AB761150
Apis mellifera SW	BK005512	*Heliconius sapho* UV2	GQ451908	*Sogatella furcifera* UV1	AB761151
Apis mellifera LW1	BK005514	*Indolestes peregrinus* RhLWA1	LC009292	*Sogatella furcifera* UV2	AB761152
Apis mellifera LW2	BK005515	*Indolestes peregrinus* RhLWA2	LC009293	*Sympetrum frequens* RhLWA1	LC009060
Apis mellifera UV	BK005513	*Indolestes peregrinus* RhLWC1	LC009294	*Sympetrum frequens* RhLWA2	LC009061
Ceratosolen solmsi SW	JX422073	*Indolestes peregrinus* RhLWD1	LC009295	*Sympetrum frequens* RhLWB1	LC009062
Ceratosolen solmsi LW1	JX422114	*Indolestes peregrinus* RhLWD2	LC009296	*Sympetrum frequens* RhLWC1	LC009063
Ceratosolen solmsi LW2	JX422151	*Indolestes peregrinus* RhLWE1	LC009297	*Sympetrum frequens* RhLWD1	LC009064
Ceratosolen solmsi UV	JX422159	*Indolestes peregrinus* RhLWE2	LC009298	*Sympetrum frequens* RhLWE1	LC009065
Danaus plexippus SW	AY605544	*Indolestes peregrinus* RhLWF1	LC009299	*Sympetrum frequens* RhLWF1	LC009066
Danaus plexippus LW	AY605545	*Indolestes peregrinus* RhSWa1	LC009300	*Sympetrum frequens* RhLWF2	LC009067
Danaus plexippus UV	AY605546	*Indolestes peregrinus* RhSWb1	LC009301	*Sympetrum frequens* RhLWF3	LC009068
Dianemobius nigrofasciatus SW	AB291232	*Indolestes peregrinus* RhUV	LC009302	*Sympetrum frequens* RhLWF4	LC009069
Dianemobius nigrofasciatus LW	FJ232921	*Laodelphax striatella* LW	AB761153	*Sympetrum frequens* RhSWa1	LC009070
Dianemobius nigrofasciatus UV	AB458852	*Laodelphax striatella* UV1	AB761154	*Sympetrum frequens* RhSWb1	LC009071
Drosophila melanogaster Rh1	Flybase: CG4550	*Laodelphax striatella* UV2	AB761155	*Sympetrum frequens* RhSWc1	LC009072
Drosophila melanogaster Rh2	Flybase: CG16740	*Macroglossum stellatarum* SW	KF539426	*Sympetrum frequens* RhSWc2	LC009073
Drosophila melanogaster Rh3	Flybase: CG10888	*Macroglossum stellatarum* LW	KF539444	*Sympetrum frequens* RhSWc3	LC009074
Drosophila melanogaster Rh4	Flybase: CG9668	*Macroglossum stellatarum* UV	KF539456	*Sympetrum frequens* RhUV	LC009075
Drosophila melanogaster Rh5	Flybase: CG5279	*Manduca sexta* SW	AD001674	*Thermonectus marmoratus* UV2	EU921225
Drosophila melanogaster Rh6	Flybase: CG5192	*Manduca sexta* LW	L78080	*Thermonectus marmoratus* UV1	EU921226
Gryllus bimaculatus SW	HM363622	*Manduca sexta* UV	L78081	*Thermonectus marmoratus* LW	EU921227
Gryllus bimaculatus green1	HM363620	*Megoura viciae* LW	AF189714	OUTGROUP *Doryteuthis pealeii* rhodopsin	AY450853
Gryllus bimaculatus green2	HM363621	*Megoura viciae* UV	AF189715	OUTGROUP *Enteroctopus dofleini* rhodopsin	X07797
Gryllus bimaculatus UV	HM363623	*Nephotettix cincticeps* SW	AB761157	OUTGROUP *Loligo forbesii* rhodopsin	X56788
Heliconius melpomene UV1	GU324678	*Nephotettix cincticeps* LW	AB761156	OUTGROUP *Sepia officinalis* rhodopsin	AF000947
Heliconius melpomene UV2	GU324679	*Nephotettix cincticeps* UV	AB761158	OUTGROUP *Todarodes pacificus* rhodopsin	X70498
Heliconius melpomene SW	GU324689				
